# MTHFD2 promotes tumorigenesis and metastasis in lung adenocarcinoma by regulating AKT/GSK‐3β/β‐catenin signalling

**DOI:** 10.1111/jcmm.16715

**Published:** 2021-06-13

**Authors:** Yangfeng Shi, Yiming Xu, Jianchang Yao, Chao Yan, Hua Su, Xue Zhang, Enguo Chen, Kejing Ying

**Affiliations:** ^1^ Department of Respiratory and Critical Medicine Sir Run Run Shaw Hospital School of Medicine Zhejiang University Hangzhou China; ^2^ Cancer Center Zhejiang University Hangzhou China; ^3^ Department of Pathology and Pathophysiology School of Medicine Zhejiang University Hangzhou China

**Keywords:** AKT, lung adenocarcinoma, miR‐30a‐3p, MTHFD2

## Abstract

Recent studies have demonstrated that one‐carbon metabolism plays a significant role in cancer development. Methylenetetrahydrofolate dehydrogenase 2 (MTHFD2), a mitochondrial enzyme of one‐carbon metabolism, has been reported to be dysregulated in many cancers. However, the specific role and mechanism of MTHFD2 in lung adenocarcinoma (LUAD) still remains unclear. In this study, we evaluated the clinicopathological and prognostic values of MTHFD2 in LUAD patients. We conducted a series of functional experiments in vivo and in vitro to explore novel mechanism of MTHFD2 in LUAD. The results showed that MTHFD2 was significantly up‐regulated in LUAD tissues and predicted poor prognosis of LUAD patients. Knockdown of MTHFD2 dramatically inhibited cell proliferation and migration by blocking the cell cycle and inducing the epithelial‐mesenchymal transition (EMT). In addition, MTHFD2 knockdown suppressed LUAD growth and metastasis in cell‐derived xenografts. Mechanically, we found that MTHFD2 promoted LUAD cell growth and metastasis via AKT/GSK‐3β/β‐catenin signalling. Finally, we identified miR‐30a‐3p as a novel regulator of MTHFD2 in LUAD. Collectively, MTHFD2 plays an oncogenic role in LUAD progression and is a promising target for LUAD diagnosis and therapy.

## 1 INTRODUCTION

Despite a decreasing trend, lung cancer still leads in term of cancer incidence and causes the most cancer‐related deaths worldwide.^1^, ^2^, ^3^ It is estimated that there will be 235 760 new cancer cases and 131 880 deaths as a result of lung cancer in the United States in 2021.^1^ Despite the substantial progressions in surgical treatment, chemotherapy, radiotherapy, tyrosine kinase inhibitors and immunotherapy in recent decades, the 5‐year survival rate of lung cancer is only 19%.^3^ Non–small cell lung cancer (NSCLC) is responsible for approximately 85% cases of lung cancer, and the most common histological subtype is lung adenocarcinoma (LUAD).^4^ The poor prognosis is mainly ascribed to cancer recurrence and metastasis. Consequently, it is urgent to identify more effective targets for diagnosis and therapy in LUAD.

One‐carbon metabolism, as a part of metabolic reprogramming, has attracted researchers’ attention to cancer in recent years.^5^, ^6^ In addition to its primary role in nucleotide synthesis, one‐carbon metabolism also plays a crucial role in the methionine cycle, methylation reactions, ATP production and redox defence.^6^, ^7^, ^8^ The reactions in one‐carbon metabolism comprise two parallel pathways in cytosolic and mitochondrial compartments. Although most of the reactions are reversible, those enzymes catalyse to the direction of thymidylate and purine synthesis during cell proliferation especially in cancer cells.^7^, ^8^ Moreover, the mitochondrial enzymes are reported to be up‐regulated in numerous cancers.^7^, ^9^, ^10^, ^11^

Methylenetetrahydrofolate dehydrogenase 2 (MTHFD2), which is known as a mitochondrial one‐carbon metabolism enzyme,^7^ is reported to be up‐regulated in various tumours.^10^, ^12^, ^13^, ^14^ Overexpression of MTHFD2 predicts poor prognosis and promotes colorectal cancer cell proliferation and metastasis.^14^ In breast cancer, MTHFD2 is identified to regulate the migration and invasion abilities of tumour cells.^10^ MTHFD2 overexpression not only facilitates cancer cell migration and invasion, but also predicts poor clinical outcomes in hepatocellular carcinoma and renal cell carcinoma.^12^, ^13^ In addition to its canonical role to facilitate one‐carbon metabolism for purine synthesis, recent studies characterize MTHFD2 with redox defence, epigenetic modification, RNA translation and DNA repair.^14^, ^15^, ^16^, ^17^ Knockdown of MTHFD2 in colorectal cancer hinders NADPH production and makes cancer cells more sensitive to oxidative stress.^14^ In renal cell carcinoma, MTHFD2 promotes the mRNA methylation of HIF‐2α via metabolic reprogramming.^16^ Moreover, MTHFD2 is found to be involved in RNA metabolism and translation by interacting with nuclear proteins in cancer cells.^17^ Besides its role in mitochondria, MTHFD2 located in the nucleus stabilizes the phosphorylation of EXO1 to promote homologous recombination repair, which ensures the pluripotency of mouse stem cells.^15^ Since MTHFD2 is present in embryonic development but barely expressed in most adult tissues,^18^ it is a promising therapeutic target. Although MTHFD2 has been reported to promote NSCLC proliferation,^19^ the underlying mechanism of MTHFD2 in LUAD is largely unknown.

miRNAs are a kind of highly conserved small non‐coding RNAs, which regulate gene expression by affecting the translation and stability of mRNAs, which modulate cell differentiation, survival and tumorigenesis.^20^ In many diseases, miRNAs show powerful regulatory ability to modulate one‐carbon metabolism.^21^, ^22^, ^23^ miR‐370 and miR‐373 are reported to alter one‐carbon metabolism via SHMT2 and MECP2 to alleviate osteoarthritis.^21^ In gastric cancer, miR‐6778‐5p strengthens stem cell signature by regulating cytosolic enzyme SHMT1.^22^ In breast cancer, MTHFD2 is identified as a target of miR‐9 that inhibits cell proliferation.^23^ Nevertheless, the relationship between miRNAs and MTHFD2 in LUAD remains to be established.

In the current study, we explored the expression, clinical characteristics and prognostic value of MTHFD2 in LUAD patients. Then, we elucidated the effect of MTHFD2 on cell proliferation, apoptosis and migration of LUAD cells in vitro and in vivo. Mechanically, we revealed that MTHFD2 was involved in AKT/GSK‐3β/β‐catenin signalling. Furthermore, we found that miR‐30a‐3p negatively regulated MTHFD2 in LUAD. Therefore, this study exhibited the clinical values of MTHFD2 in LUAD patients and determined the biological effects, potential molecular mechanism and regulation of MTHFD2 in LUAD cells.

## 2 MATERIALS AND METHODS

### 2.1 Patient tissue collection

This study was permitted by the ethical committee of Sir Run Run Shaw Hospital. A total of 21 paired primary cancer tissues and adjacent normal lung tissues were collected from LUAD patients in Sir Run Run Shaw Hospital from January 2019 to January 2020. Written informed consents were obtained from each patient, who had not received any radiotherapy or chemotherapy before surgery. The normal lung tissues were cut at least 5 cm away from cancer tissues. All samples were histologically confirmed by pathologists. The fresh tissues were instantly frozen in liquid nitrogen and then stored at −80℃, or fixed and paraffin‐embedded.

### 2.2 Cell culture and reagents

The LUAD cell lines (H322, SPC‐A‐1, PC‐9, H1299, H1975, A549, H1650, HCC827), NSCLC cell lines (H460, SK‐MES‐1) and HEK293T cells were purchased from American Type Culture Collection (ATCC). Human bronchial epithelial cell (HBEC) was a gift from School of Medicine, Zhejiang University. The LUAD cell lines, NSCLC cell lines and HBEC were maintained in RPMI 1640 medium (Solarbio). HEK293T cells were maintained in DMEM (Solarbio, China). All medium were complemented with 10% foetal bovine serum (FBS, Noverse, 100 units/mL penicillin and 100 μg/mL streptomycin (HyClone). All cells were maintained in a humidified incubator containing 5% CO_2_ at 37℃. An AKT inhibitor MK‐2206 was purchased from Meilunbio.

### 2.3 Cell transfection and lentivirus infection

For transient transfection, cells were seeded into the 6‐well plates and grew to 70% confluence before transfection. To prepare transfection complexes, 125 μL Opti‐MEM (Gibco) with 50 nM siRNA or miRNA mimics and 125 μL Opti‐MEM with 1000 ng plasmid were prepared for each well. Next, cells were transfected following the manufacturer's protocol of Lipofectamine 3000 (L3000015, Invitrogen). The medium was refreshed after transfection for 4‐6 hours. Cells were applied to other assays after transfection for 24‐48 hours.

For stable transfection, cells were seeded into the 24‐well plates and reached 50% confluence before transfection. The lentivirus (1:100) packaged with short hairpin RNA (shRNA) and polybrene (1:1000) was added to cells. After 48 hours, puromycin (1 μg/mL, Beyotime) was applied to select stably infected cells for 2‐7 days.

The siRNAs, miRNA mimics and lentivirus LV2 (U6/Puro) packaged shRNAs were purchased from Genepharma. The sequences were listed as follows: siMTHFD2#1, sense 5′‐GCGAGAAUCCUGCAAGUCATT‐3′; siMTHFD2#2, sense 5′‐ GCCUCUUCCAGAGCAUAUUTT‐3′; miR‐30a‐3p mimics, sense 5′‐ CUUUCAGUCGGAUGUUUGCAGC‐3′; lentivirus‐shMTHFD2, 5′‐ CGAATGTGTTTGGATCAGTAT‐3′. The MTHFD2 overexpression plasmid was synthesized by Genepharma as pEX‐3 ‐MTHFD2.

### 2.4 Real‐time quantitative PCR (RT‐qPCR)

RNA was extracted using RNA extract reagent according to the manufacturer's protocol (AP‐MN‐MS‐RNA‐250, Axygen). HiFiScript cDNA Synthesis Kit (CW2569, CWBIO) and miRNA cDNA Synthesis Kit (CW2141, CWBIO) were applied to reverse transcribe mRNA and miRNA into cDNA, respectively. Then, SYBR Premix Ex Taq^™^ II (RR820A, Takara) was applied to perform RT‐qPCR in LightCycler480 system (Roche) with two steps. β‐Actin was used as the internal control to calculate the relative mRNA levels of target genes using the 2^‐△△CT^ method. The primers synthesized by TSINGKE were as follows: MTHFD2 (F: 5′‐GATCCTGGTTGGCGAGAATCC‐3′, R: 5′‐TCTGGAAGAGGCAACTGAACA‐3′), β‐Actin (F: 5′‐GGCATCCTCACCCTGAAGTA‐3′, R: 5′‐GGGGTGTTGAAGGTCTCAAA‐3′), miR‐30a‐3p (F: 5′‐AAGGCGGCTTTCAGTCGGATGTT‐3′), U6 (F: 5′‐CTCGCTTCGGCAGCACA‐3′), universal R (R: 5′‐GGCCAACCGCGAGAAGATG‐3′).

### 2.5 Western blot

Total protein was extracted using RIPA lysis buffer (Beyotime) containing phosphatase and protease inhibitors (Solarbio). Nuclear protein was extracted using Nuclear Protein Extraction Kit (Solarbio, R0050). Protein concentration was detected using BCA protein assay kit (Beyotime). Equal amount of protein (10 μg) was loaded onto 10% SDS‐PAGE and then transferred to PVDF membranes (Bio‐Rad, USA). All membranes were then blocked for 1 hour with 5% BSA and incubated at 4℃ overnight with primary antibodies. Next day, the membranes were incubated at room temperature for 2 hours with HRP‐conjugated secondary antibodies (CST, #7076, #7074, 1:1000). Protein signals were visualized with ECL detection kit (FDbio). The relative protein levels were analysed by ImageJ software. β‐Actin was used as the internal control. Primary antibodies were as follows: MTHFD2 (Abcam, ab151447, 1:3000), β‐Actin (CST, #8457, 1:1000), E‐cadherin (CST, #3195, 1:1000), N‐cadherin (CST, #13116, 1:1000), Vimentin (CST, #5741, 1:1000), Erk1/2 (CST, #4695, 1:1000), p‐Erk1/2 (CST, #4370, 1:2000), Akt (CST, #4691, 1:1000), p‐Akt (CST, #4060, 1:2000), GSK‐3β (Abcam, ab32391, 1:5000), p‐GSK‐3β (Abcam, ab75814, 1:10 000) and β‐catenin (CST, #8480, 1:1000).

### 2.6 CCK‐8 cell viability assay

After transfection, cells were seeded at a dense of 2000 cells into 96‐well plates per well. Each well was added 10 μL CCK‐8 reagent (APExBio, USA) and incubated at 0, 1, 2, 3 and 4 days for 2 hours. Absorbance (OD) at 450 nm was used to access the cell viability.

### 2.7 Cell cycle and cell apoptosis

For cell cycle analysis, 75% cold ethanol was used to fix cells and cells were kept at 4℃ over night. Next day, propidium iodide (PI)/RNase Staining Buffer (550825, BD Biosciences) was applied to stain the cells. Then, FACS Calibur (BD Biosciences) was used for analysis. Cell cycle distributions were quantified by ModFit LT software.

For cell apoptosis, Annexin V‐FITC/PI binding buffer (C1062L, Beyotime) was applied to stain the cells. Then, FACS Calibur (BD Biosciences) was used for analysis. The percentages of apoptotic cells were quantified by FlowJo software.

### 2.8 Wound healing assay and transwell assay

For wound healing assay, 24‐well plates were applied and cells were incubated until 90% confluence. Scratch wounds were made with 1‐mL sterile pipette tips across cell surface. Images of wound closure were collected at 0 hour and 24 hours using a microscope.

For transwell migration assay, 200 μL FBS‐free medium with 1 × 10^5^ cells was added into upper chambers of Transwell chambers (3422, Corning). Lower chambers were filled with 600 μL 10% FBS medium. After incubating for 24‐48 hours, methanol and 0.25% crystal violet (Solarbio) were applied to fix and stain the cells. Microscope was used to count the stained cells, and the mean numbers of cells in three random fields were calculated as the results.

### 2.9 Dual luciferase reporter assay

HEK293T cells were plated into 24‐well plates to reach 50% confluence before transfection. Then, HEK293T cells were co‐transfected with 20 ng Renilla plasmid, 200 ng target plasmid and 50 nM NC or miR‐30a‐3p mimics using Lipofectamine 3000 (L3000015, Invitrogen) each well. After transfection for 48 hours, cells were lysed. Dual‐Lumi^™^ II Luciferase Assay Kit (RG089S, Beyotime) was applied to measure Firefly and Renilla luciferase activities. The firefly‐luciferase plasmids CV045‐MTHFD2‐3′UTR‐WT and CV045‐MTHFD2‐3′UTR‐MUT were synthesized by Genechem.

### 2.10 Immunohistochemistry (IHC) and TUNEL assay

Tissues were fixed and embedded in paraffin. Tissue sections were stained with haematoxylin and eosin (HE), or incubated with primary antibodies (MTHFD2, Abcam, ab151447, 1:500; Ki67, Abcam, ab92742, 1:1000). The protein levels were detected using DAB staining kit (Beyotime). IHC scores were calculated on the basis of both the proportion of stained cells (1, 0%‐25%; 2, 25%‐50%; 3, 50%‐75%; 4, 75%‐100%) and the intensity of staining (0, none; 1, light yellow; 2, yellow; 3, brown). IHC scores determined high (>4) or low (<4) protein levels by multiplying the proportion and the intensity. One Step TUNEL Apoptosis Assay Kit (Beyotime) was used to perform TUNEL assay.

### 2.11 Tumorigenesis and metastasis in vivo

The Institutional Animal Care and Use Committee of Zhejiang University approved all experimental procedures. Male BALB/c‐nude mice (4 weeks old) were housed in specific pathogen‐free environment and randomly divided into 5 mice per group.

For subcutaneous xenograft tumour model, stably transfected cells (2 × 10^7^/mouse) were resuspended in 50% Matrigel matrix (354234, Corning) and injected into the nude mice subcutaneously. Tumour sizes were measured using vernier calliper every three days. The tumour volumes were calculated on the basis of the formula: volume = length × width^2^/2. After injection, Mice were killed for weighing the tumour and IHC staining after 3 weeks.

For tail vein metastatic tumour model, stably transfected cells (2 × 10^6^/mouse) were resuspended in PBS and injected into the nude mice through tail veins. Six weeks after injection, mice were killed for removing the lungs and HE staining. The metastatic tumours were counted using a microscope.

### 2.12 Statistical analysis

All experiments had three individual times repetition. All statistical analyses were conducted using GraphPad Prism 7 software and SPSS 20.0. The difference between two groups was compared using Student's *t* test. One‐way ANOVA or two‐way ANOVA was used to compare multiple groups. The results were shown as mean ± standard deviation (SD). Overall survival was compared using log‐rank test and presented as Kaplan‐Meier survival curve. GSEA analysis was performed based on MTHFD2 expression of TCGA LUAD patients. The correlation between two groups was analysed using Pearson's correlation test. *P* < .05 was set as statistical significance.

## 3 RESULTS

### 3.1 MTHFD2 is up‐regulated in LUAD patients and predicts poor prognosis

**FIGURE 1:**
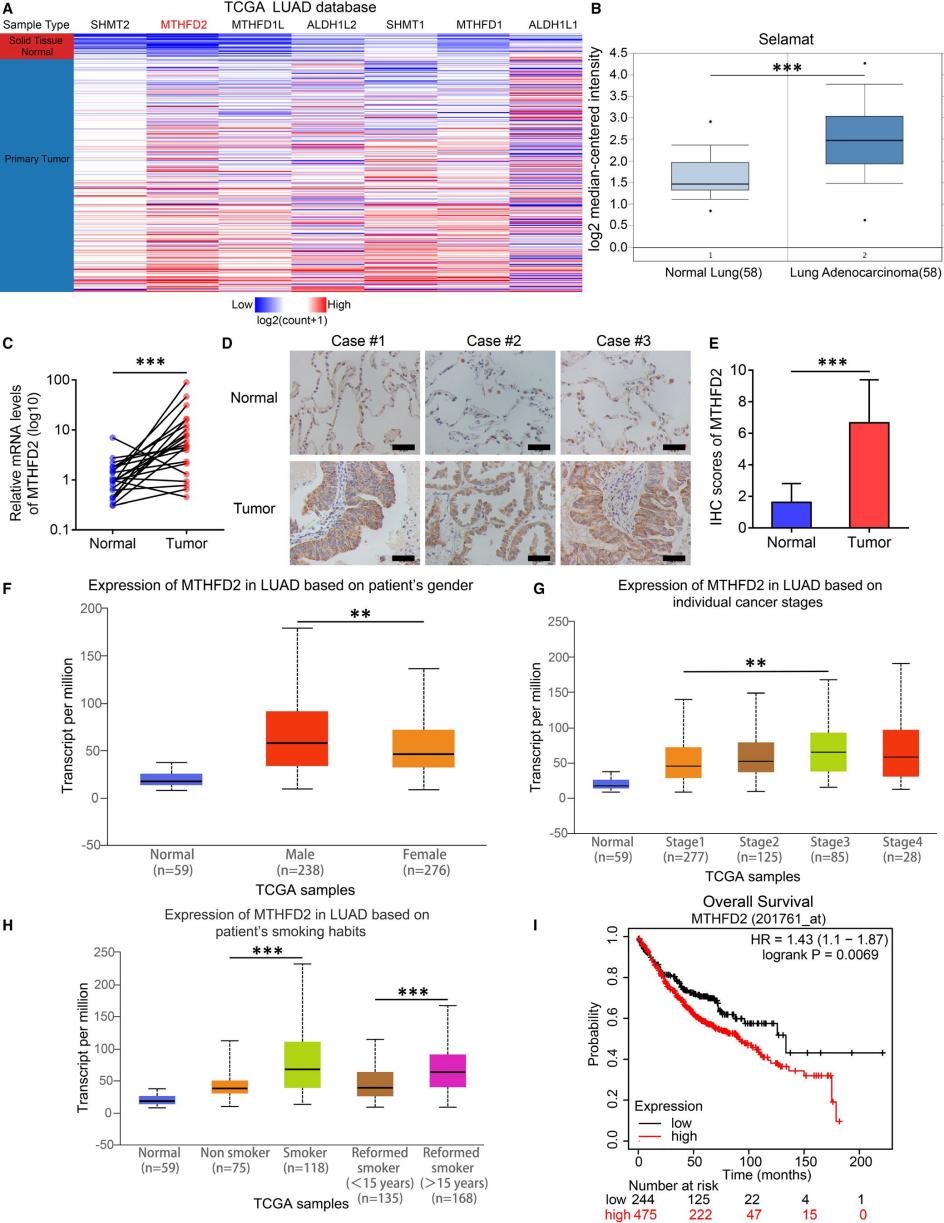
The expression and clinical characteristics of MTHFD2 in LUAD. A, Expression profiles of enzymes that involved in cytoplasmic and mitochondrial one‐carbon metabolism pathways based on TCGA LUAD samples using UCSC Xena. B, The relative mRNA expression levels of MTHFD2 in Selamat LUAD samples compared with normal lung tissues from Oncomine Database. C, The relative mRNA levels of MTHFD2 in 21 paired normal lung and tumour tissues from LUAD patients. D and E, The representative IHC staining and scores of MTHFD2 in 21 paired normal lung and tumour tissues from LUAD patients. Scale bar, 50 μm. F, G and H, Differential expression of MTHFD2 based on patient's gender, individual cancer stages, patient's smoking habits in LUAD patients from TCGA database using UALCAN. I, Prognostic analysis for overall survival (OS) of LUAD patients stratified by MTHFD2 expression level using Kaplan‐Meier plotter. *P < .05, **P < .01, ***P < .001

Since one‐carbon metabolism enzymes are reported to be dysregulated in cancers,^7^, ^24^ especially those in the mitochondrial pathway, we investigated their expression in The Cancer Genome Atlas (TCGA) Lung Adenocarcinoma (LUAD) samples using UCSC Xena.^25^ The mRNA levels of the enzymes, especially the mitochondrial enzymes, were obviously up‐regulated in LUAD tissues (Figure 1A), and MTHFD2 was the most overexpressed gene. In addition to TCGA database, overexpression of MTHFD2 was also supported by the data from Oncomine Database (Figure 1B). Furthermore, the mRNA level and protein level of MTHFD2 in clinical LUAD tissues were also notably elevated compared with 21 paired normal lung tissues according to qPCR (Figure 1C) and immunohistochemistry (IHC) staining (Figure 1D,E). In addition, the expression level of MTHFD2 was higher in male patients than female patients and was positively associated with smoking habit, smoking years and individual cancer stage (Figure 1F‐H) based on TCGA LUAD samples using UALCAN.^26^ Kaplan‐Meier plotter^27^ showed that the overall survival time of LUAD patients with higher MTHFD2 expression was shorter than those with lower MTHFD2 expression (Figure 1I). Overall, MTHFD2 is strongly associated with LUAD and has notable clinical values.

### 3.2 MTHFD2 knockdown inhibits LUAD cell proliferation and induces LUAD cell apoptosis

**FIGURE 2:**
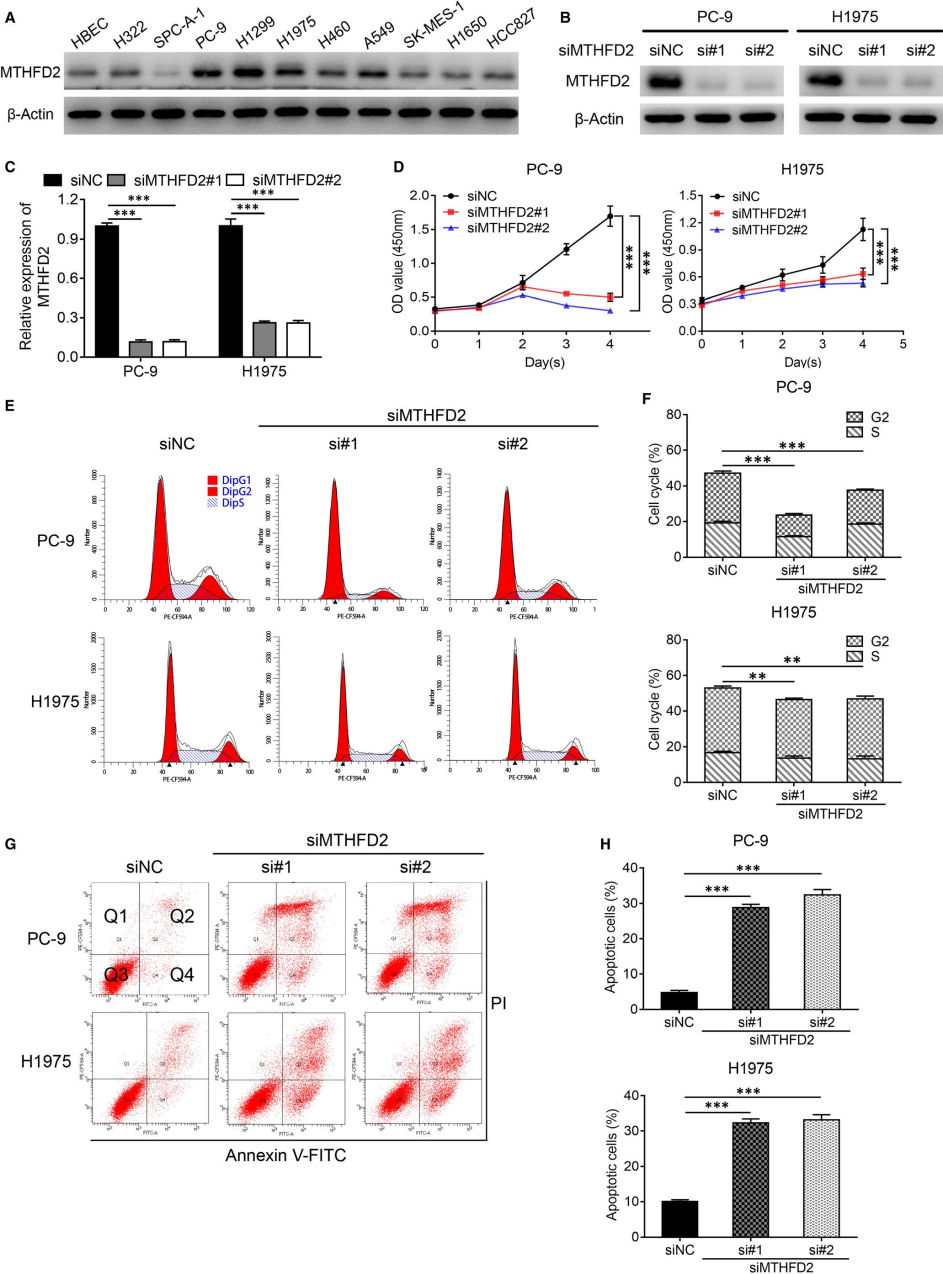
Knockdown of MTHFD2 inhibits proliferation and promotes apoptosis in LUAD cells. A, Western blot analysis for MTHFD2 expression in NSCLC cell lines and human bronchial epithelial cells (HBECs). B and C, Western blot confirmed the knockdown efficiency of MTHFD2 in PC‐9 and H1975 cells with siRNA (#1, #2). siNC, negative control siRNA. D, CCK‐8 assay assessed the proliferation of PC‐9 and H1975 cells after transfection. E and F, The effect of MTHFD2 knockdown on cell cycle in PC‐9 and H1975 cells as indicated treatment for 48 h. G and H, Cell apoptosis (Q2 + Q4) of PC‐9 and H1975 were measured using Annexin Ⅴ‐FITC/PI staining after transfection with siNC or siMTHFD2 (si#1, si#2) for 48 h. *P < .05, **P < .01, ***P < .001

Given the MTHFD2 overexpression found in LUAD tissues, we tested its expression in LUAD cell lines. As shown by Western blot, MTHFD2 protein level was basically higher in LUAD and two other NSCLC cell lines than in HBEC (Figure 2A). To clarify the role of MTHFD2 in LUAD, MTHFD2 was knocked down using small interfering RNAs (siRNAs) in PC‐9 and H1975 cells and overexpressed using plasmid in SPC‐A‐1 cells. Then, Western blot confirmed the knockdown efficiency and overexpression efficiency (Figure 2B,C, Figure S1A). The CCK‐8 assay demonstrated that knockdown of MTHFD2 inhibited cell proliferation in PC‐9 and H1975 cells while overexpression of MTHFD2 promoted cell proliferation in SPC‐A‐1 cells (Figure 2D, Figure S1B). Flow cytometry analysis manifested that MTHFD2 knockdown decreased the proportions of S and G2 phases in LUAD cells (Figure 2E,F) while MTHFD2 overexpression had reverse effect on SPC‐A‐1 cells (Figure S1C,D). Moreover, MTHFD2 knockdown induced obvious cell death (Figure 2G,H). Taken together, MTHFD2 overexpression promotes LUAD growth.

### 3.3 MTHFD2 knockdown suppresses LUAD cell migration

**FIGURE 3:**
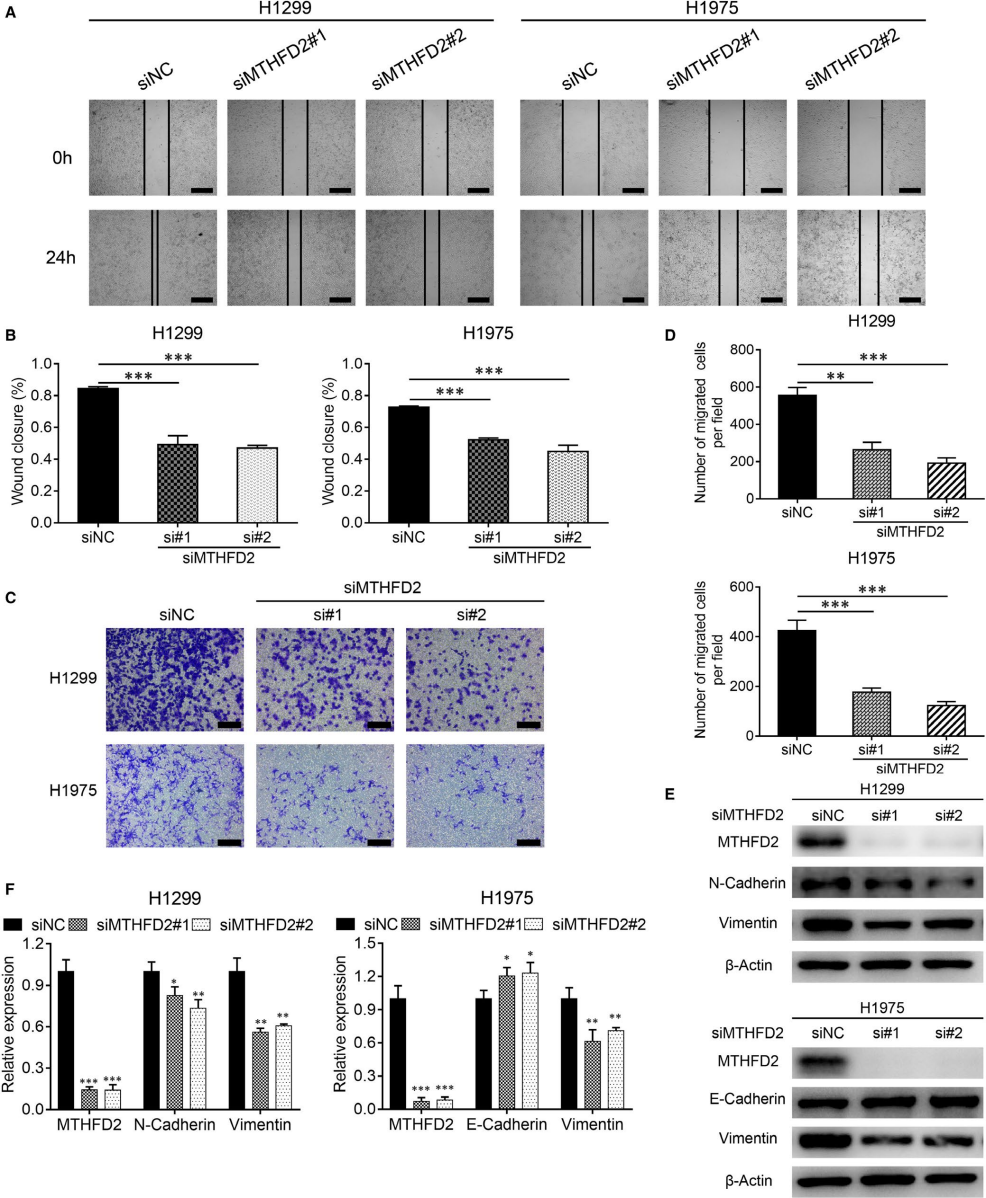
Knockdown of MTHFD2 inhibits migration ability of LUAD cells. A and B, Wound healing assay showed the migration ability of H1299 and H1975 cells after transfection. Scale bar, 500 μm. C and D, Representative images of transwell assay in H1299 and H1975 cells after transfection. Scale bar, 200 μm. E and F, Western blot analysis for N‐cadherin and vimentin expression of H1299 cells, E‐cadherin and vimentin expression of H1975 cells as indicated treatment for 48 h. *P < .05, **P < .01, ***P < .001

We next assessed the impact of MTHFD2 on the migration capacity of LUAD cells. The wound healing assay and transwell assay showed that knockdown of MTHFD2 obviously limited H1299 and H1975 cell migration (Figure 3A‐D) while overexpression of MTHFD2 enhanced SPC‐A‐1 cell migration (Figure S2A‐D). Since epithelial‐mesenchymal transition (EMT) serves as a well‐known mechanism involved in cancer metastasis,^28^ we then investigated the alteration of common EMT markers by MTHFD2. As shown in H1299 and H1975 cells, MTHFD2 knockdown notably decreased the expression of vimentin and N‐cadherin, while increased the expression of E‐cadherin (Figure 3E,F). On the contrary, MTHFD2 overexpression increased the expression of N‐cadherin and decreased the expression of E‐cadherin (Figure S2E,F).

### 3.4 MTHFD2 knockdown suppresses the tumorigenesis and metastasis of LUAD cells in vivo

**FIGURE 4:**
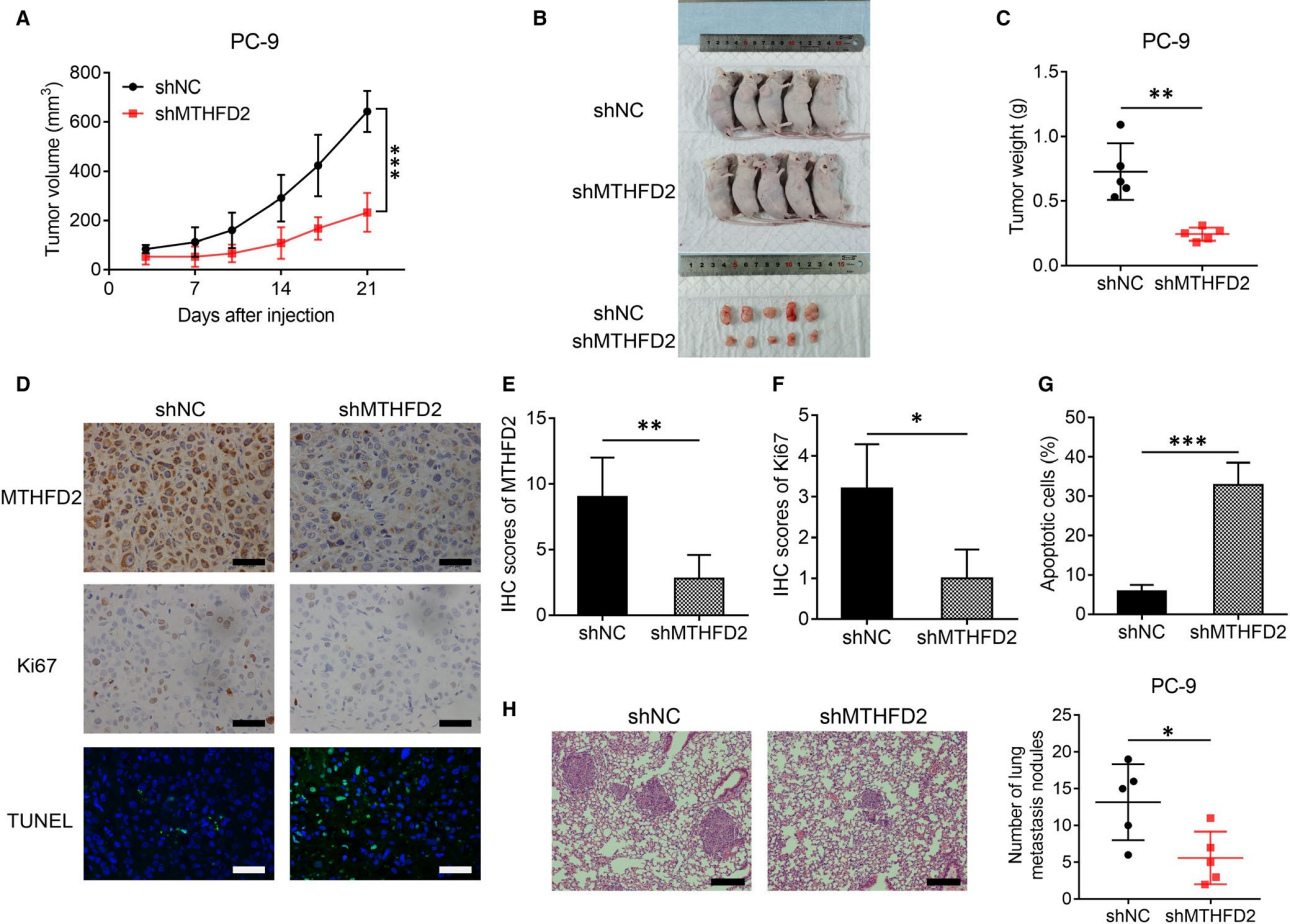
MTHFD2 knockdown inhibits LUAD tumorigenesis and metastasis in nude mice. A, Tumour growth curves were recorded from established xenograft model of stable MTHFD2 knockdown or negative control PC‐9 cells. shMTHFD2, stable MTHFD2 knockdown. shNC, negative control. B, Photograph of xenograft model and dissected tumours. C, Tumour weight of the dissected tumours. D, E, F and G, The representative staining and scores of MTHFD2, Ki67 and TUNEL from xenograft tumours. Scale bar, 50 μm. H, Metastatic nodules in the lungs of nude mice tail vein model (three sections evaluated per lung). Scale bar, 200 μm. *P < .05, **P < .01, ***P < .001

To further confirm the impact of MTHFD2 on LUAD tumorigenesis in vivo, PC‐9 cells with stable MTHFD2 knockdown (shMTHFD2) were injected into nude mice subcutaneously. In accordance with the results in vitro, the shMTHFD2 PC‐9 group grew much slower than the shNC PC‐9 group (Figure 4A). The shMTHFD2 PC‐9 group showed decreased tumour volume and weight compared with the shNC PC‐9 group (Figure 4B,C). In addition, the shMTHFD2 PC‐9 group exhibited a notably reduced cell proliferation index and increased cell apoptosis, as determined by Ki‐67 and TUNEL staining (Figure 4D‐G). The tail vein metastatic tumour assay showed that mice injected with shMTHFD2 PC‐9 cells had significantly fewer metastatic nodules in the lung than those injected with shNC PC‐9 cells (Figure 4H).

### 3.5 MTHFD2 knockdown inhibits the AKT/GSK‐3β/β‐catenin signalling to suppress LUAD proliferation and metastasis

**FIGURE 5:**
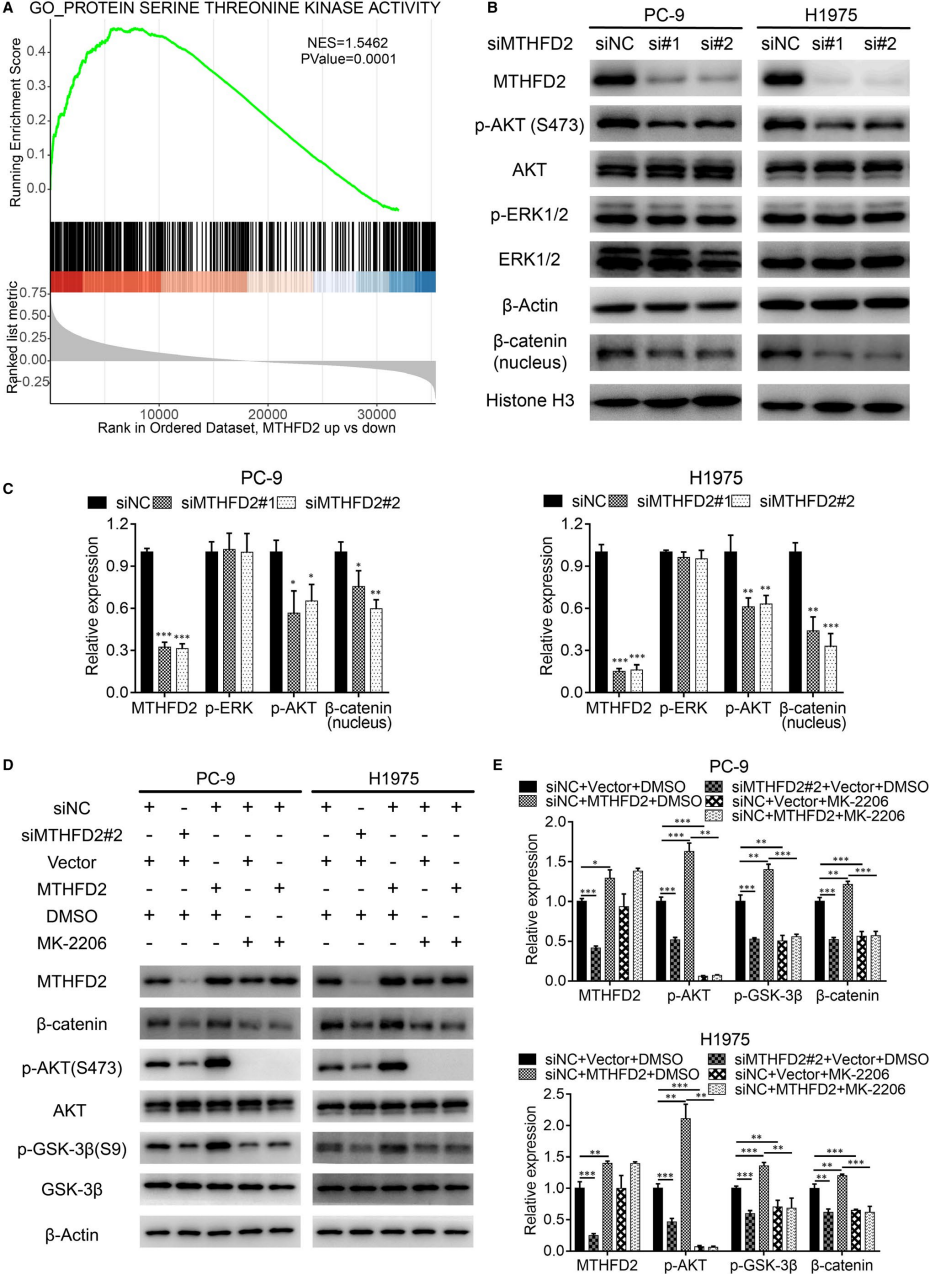
MTHFD2 knockdown inhibits the AKT/GSK‐3β/β‐catenin signalling pathway. A, GSEA revealed high MTHFD2 expression was associated with protein serine threonine kinase activity gene set based on TCGA LUAD samples. NES, normalized enrichment score. B and C, Western blot analysis for p‐AKT (S473), AKT, p‐ERK1/2, ERK1/2 and nuclear β‐catenin expression of PC‐9 and H1975 cells after transfection. D and E, Western blot analysis for p‐AKT (S473), AKT, p‐ GSK‐3β (S9), GSK‐3β, β‐catenin and MTHFD2 expression of PC‐9 and H1975 cells after transfection with siNC or si#2 (48 h), vector or MTHFD2 plasmid (24 h) in the presence of DMSO or MK‐2206 (10 μM, 24 h) as indicated. *P < .05, **P < .01, ***P < .001

**FIGURE 6:**
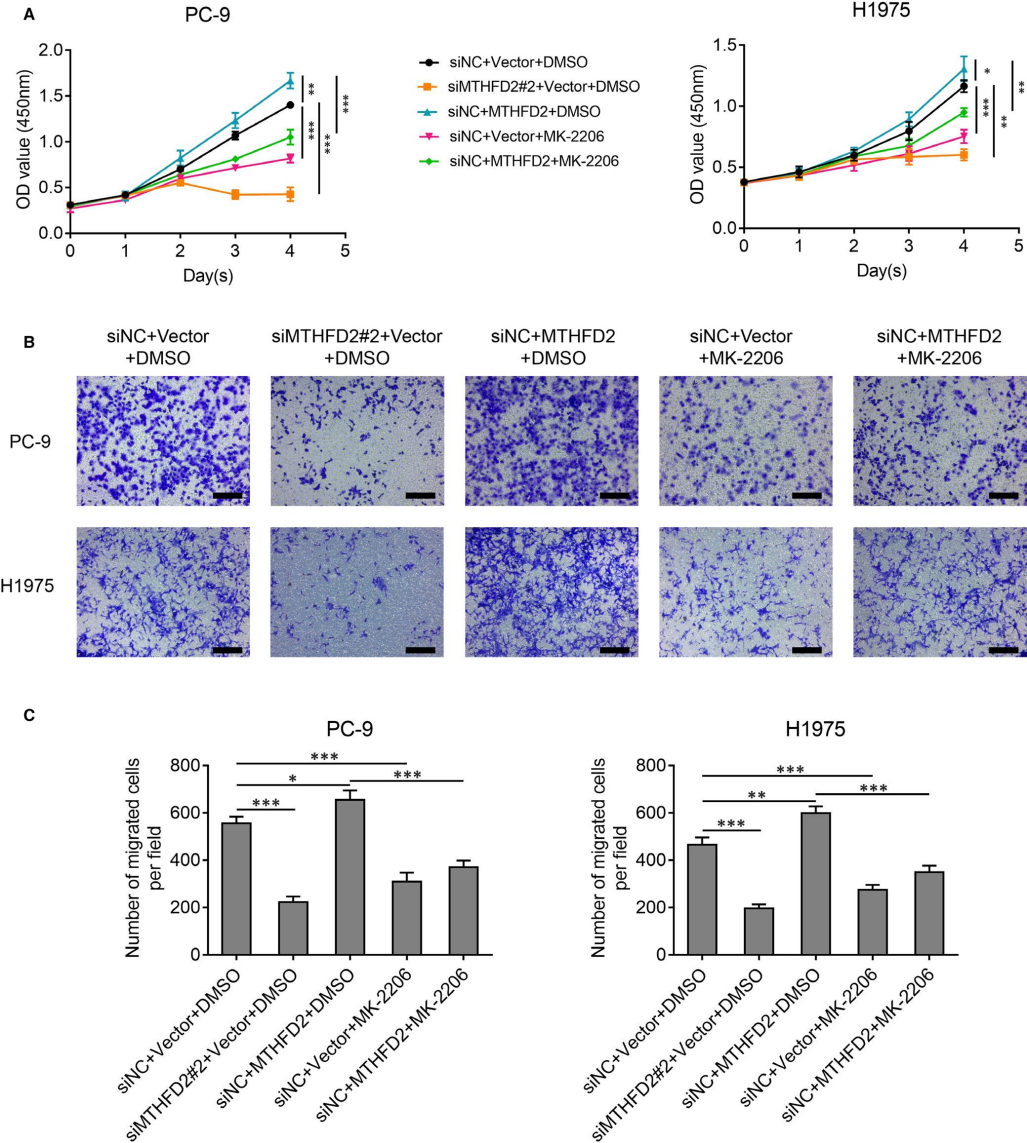
MTHFD2 regulates LUAD cell proliferation and migration via AKT signalling. A, CCK‐8 assay assessed the proliferation of PC‐9 and H1975 cells for 4 d as the indicated treatment. B and C, Transwell assay showed the migration ability of PC‐9 and H1975 cells after the indicated treatment for 48 h. Scale bar, 200 μm. *P < .05, **P < .01, ***P < .001

To further elucidate the mechanism of MTHFD2 in LUAD development, we performed gene set enrichment analysis (GSEA) of MTHFD2 on TCGA LUAD samples. We noticed that MTHFD2 could affect potential protein serine/threonine kinase activity (Figure 5A). Western blot analysis showed that MTHFD2 knockdown had no effect on ERK1/2 signalling, but significantly decreased AKT phosphorylation at Ser473 in PC‐9 and H1975 cells (Figure 5B,C). Since MTHFD2 regulated LUAD migration via EMT, reduced nuclear β‐catenin was further confirmed with MTHFD2 knockdown (Figure 5B,C). GSK3β serves as a downstream effector of AKT and mediates the degradation of β‐catenin via phosphorylation.^29^, ^30^ We wondered whether GSK3β also participated in the oncogenic activity of MTHFD2. Interestingly, we discovered reduced phosphorylation of AKT at Ser473 and of GSK3β at Ser9 along with β‐catenin suppression in MTHFD2 knockdown PC‐9 and H1975 cells, which occurred after MK‐2206 treatment (an efficient AKT allosteric inhibitor) (Figure 5D,E). On the contrary, MTHFD2 overexpression in PC‐9 and H1975 cells obviously elevated the expression level of β‐catenin and the phosphorylation of AKT/GSK3β, effects that were abrogated by MK‐2206 treatment (Figure 5D,E). Given that MTHFD2 could activate AKT/GSK3β/β‐catenin signalling in LUAD cells, we assumed that this signalling was involved in the promotion of LUAD by MTHFD2. As shown by CCK‐8 and transwell assay in Figure 6A‐C, MTHFD2 knockdown inhibited LUAD cell proliferation and migration. MTHFD2 overexpression conversely enhanced LUAD cell proliferation and migration, while MK‐2206 erased this effect. Overall, these findings demonstrate that MTHFD2 can promote LUAD via the AKT/GSK‐3β/β‐catenin signalling.

### 3.6 MTHFD2 overexpression is mediated by down‐regulation of miR‐30a‐3p in LUAD cells

**FIGURE 7:**
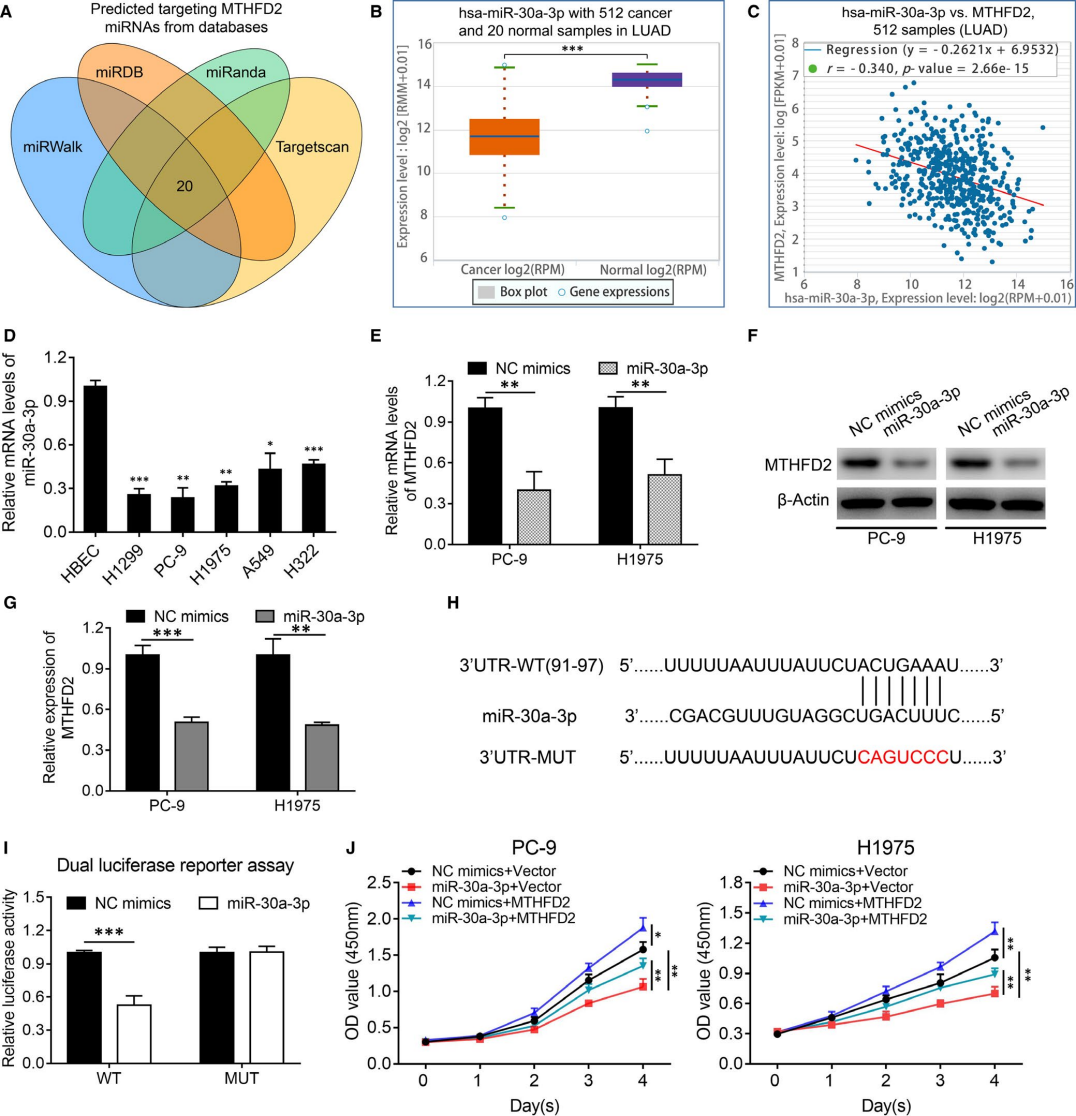
MTHFD2 overexpression is mediated by down‐regulation of miR‐30a‐3p in LUAD cells. A, The common miRNAs targeting MTHFD2 were predicted by the miRWalk, miRDB, miRanda and TargetScan Databases. B, The relative expression levels of miR‐30a‐3p in LUAD samples compared with normal lung tissues from TCGA database using starBase. C, Correlation analysis of relationship between MTHFD2 and miR‐30a‐3p mRNA expression levels in LUAD samples from TCGA database using starBase. D, RT‐qPCR analysis of miR‐30a‐3p mRNA expression levels in LUAD cell lines compared with HBECs. E, F and G, RT‐qPCR and western blot analysis for MTHFD2 mRNA and protein levels in PC‐9 and H1975 cells after transfection with NC mimics or miR‐30a‐3p mimics. H, Schematic of miR‐30a‐3p putative binding site in the wild‐type (WT) and mutant (MUT) 3′UTR of MTHFD2. I, The dual luciferase reporter assay determined the interaction between miR‐30a‐3p and 3′UTR of MTHFD2. J, CCK‐8 assay assessed the viability of PC‐9 and H1975 cells after co‐transfected with NC mimics or miR‐30a‐3p mimics, and vector or MTHFD2 plasmid. *P < .05, **P < .01, ***P < .001

We further explored why MTHFD2 is up‐regulated in LUAD. Since miRNAs play important roles in tumorigenesis mainly through regulating genes including one‐carbon metabolism enzymes via post‐transcriptional processes, we next explored potential miRNAs targeting MTHFD2. Twenty common candidate miRNAs were screened from the miRWalk, miRDB, miRanda and TargetScan databases (Figure 7A, Table S1). MiR‐30a‐3p was identified from them because it was reported to be down‐regulated in many cancers, including NSCLC.^31^, ^32^, ^33^ The results derived from TCGA LUAD samples using starBase^34^ showed that LUAD tissues had much lower miR‐30a‐3p expression level compared with normal lung tissues (Figure 7B), and miR‐30a‐3p exhibited a negative relationship to MTHFD2 (Figure 7C). In line with the results from the database, we confirmed LUAD cells also had lower miR‐30a‐3p expression levels than in HBECs (Figure 7D). In addition, transfection with miR‐30a‐3p mimics significantly inhibited MTHFD2 at both mRNA and protein levels in LUAD cells (Figure 7E‐G). Dual luciferase reporter assay validated that miR‐30a‐3p interacted with MTHFD2 at sites 91‐97 of the 3′UTR (Figure 7H,I). Moreover, miR‐30a‐3p showed an inhibitory effect on PC‐9 and H1975 cell growth, which could be partially reversed by MTHFD2 (Figure 7J). Collectively, the down‐regulation of miR‐30a‐3p is found to be responsible for MTHFD2 overexpression in LUAD.

## 4 DISCUSSION

Metabolic reprogramming is a fundamental property of tumorigenesis.^35^ MTHFD2, a mitochondrial one‐carbon metabolism enzyme, is dysregulated in a variety of cancers.^10^, ^12^, ^14^ This study displayed the gene expression levels of one‐carbon metabolism enzymes and found that MTHFD2 was the most up‐regulated enzyme in LUAD. It has been established that NSCLC patients who smoke have a higher chance of Kras mutation than those who do not smoke,^36^ and Kras mutation is known to be associated with MTHFD2 expression via MYC transcriptional regulation.^14^, ^37^, ^38^ Both of these layers of evidence substantiate our result that male sex and smoking habit contributed to MTHFD2 expression in LUAD. Moreover, expression of MTHFD2 was positively associated with tumour stage and negatively associated with survival time in LUAD patients. These observations suggest that MTHFD2 has a crucial role in LUAD development.

Overexpression of MTHFD2 in LUAD samples implies its potent biological effect on LUAD development. We conducted a series of functional experiments on LUAD cells in vivo and in vitro. Knockdown of MTHFD2 in PC‐9 and H1975 cells decreased cell proliferation and enhanced cell apoptosis. In addition, MTHFD2 suppression exhibited an inhibitory effect on cell‐derived xenografts. Moreover, MTHFD2 knockdown blocked the cell cycle in LUAD cells at G0/G1 phase. In line with our results, MTHFD2 has been shown to co‐express with cell cycle–related proteins,^19^ as well as to promote tumour growth in hepatocellular carcinoma and renal cell carcinoma.^12^, ^13^ Metastasis is another major element responsible for cancer deaths. Accordingly, we investigated the role of MTHFD2 in LUAD metastasis. MTHFD2 suppression significantly inhibited H1299 and H1975 cell migration. Besides, MTHFD2 knockdown diminished N‐cadherin and vimentin expression in LUAD cells since EMT is a fundamental property for cancer metastasis. Since PC‐9 cells barely showed N‐cadherin and vimentin expression in our experiment conditions (data not shown), we selected H1299 and H1975 cells for migration assays. Consistently, MTHFD2 has also been reported to affect cancer cell migration and invasion abilities by modulating vimentin expression in breast cancer and renal cell carcinoma.^10^, ^13^ Furthermore, we confirmed that MTHFD2 knockdown decreased metastatic nodules from transplanted LUAD cells in cell‐derived xenografts. Taking all these into account, we demonstrated MTHFD2 facilitated growth and metastasis in LUAD.

AKT, a serine/threonine kinase, is involved in diverse biological processes, including cell survival, differentiation and motility.^39^, ^40^ AKT can inactivate GSK‐3β, a glycogen synthase and serine/threonine kinase, via phosphorylation at Ser9, which contributes to tumorigenesis.^41^ Wnt/β‐catenin signalling is known to be associated with EMT, metastasis and CSC property maintenance.^42^, ^43^ GSK‐3β‐mediated phosphorylation of β‐catenin leads to its ubiquitination and degradation.^44^ Activation of AKT/GSK‐3β signalling can lead to the accumulation and translocation of β‐catenin, which facilitates cancer malignancy.^43^, ^45^ We speculated that MTHFD2 affected protein serine/threonine kinase activity given our GSEA results. Intriguingly, we found a positive relationship between MTHFD2 and AKT signalling. Subsequently, we first revealed that AKT/GSK‐3β/β‐catenin signalling induced by MTHFD2 was involved in LUAD progression. However, AKT was reported to regulate MTHFD2 expression via c‐Myc in colorectal cancer.^14^ Based on both evidence, there may be a close connection between MTHFD2 and AKT signalling.

Accumulating evidence defines non‐coding RNAs’ pivotal role in carcinogenesis. miRNAs, a kind of small endogenous single‐stranded RNAs, consist of about 20 nucleotides, which mostly serve as negative regulators by targeting the 3′UTR of target genes at the post‐transcriptional level.^46^, ^47^ Recent studies show miRNAs can affect cancer progression via manipulating MTHFD2. Through microarray profiling, MTHFD2 is identified as a target of miR‐9 which inhibits cell proliferation in breast cancer.^23^ In acute myeloid leukaemia, miR‐92a and miR‐504‐3p serve as tumour suppressors by regulating MTHFD2.^48^, ^49^ Moreover, miR‐940 and miR‐33a‐5p are reported to inhibit cancer progression through down‐regulating MTHFD2 in glioma and colorectal cancer, respectively.^50^, ^51^ Herein, we determined that miR‐30a‐3p was down‐regulated and acted as a tumour suppressor in LUAD. Besides, we first validated that miR‐30a‐3p was a negative regulator of MTHFD2. Therefore, miR‐30a‐3p down‐regulation accounts for the overexpression and effect of MTHFD2 in LUAD.

In conclusion, our study elaborates the clinical and prognostic values of MTHFD2 in LUAD. MTHFD2 promotes LUAD progression by regulating AKT/GSK‐3β/β‐catenin signalling. Overexpression of MTHFD2 relies on miR‐30a‐3p down‐regulation in LUAD. Accordingly, MTHFD2 will become a promising target for LUAD diagnosis and therapy in the future.

## CONFLICT OF INTEREST

All authors declare no conflicts of interest.

## AUTHOR CONTRIBUTION

**Yangfeng Shi:** Conceptualization (equal); Data curation (equal); Formal analysis (equal); Investigation (equal); Methodology (equal); Writing‐original draft (lead). **Yiming Xu:** Data curation (equal); Formal analysis (equal); Methodology (equal). **Jianchang Yao:** Formal analysis (equal); Methodology (equal); Writing‐original draft (supporting). **Chao Yan:** Investigation (equal); Software (equal); Validation (equal). **Hua Su:** Data curation (supporting); Investigation (equal); Validation (equal). **Xue Zhang:** Software (equal); Supervision (equal); Writing‐review & editing (equal). **Enguo Chen:** Supervision (equal); Writing‐review & editing (equal). **Kejing Ying:** Conceptualization (equal); Funding acquisition (lead); Project administration (lead); Supervision (lead); Writing‐review & editing (equal).

## Supporting information

Fig S1

Fig S2

Table S1

## Data Availability

All data used in the current study are available from the corresponding author on reasonable request.
