# Supplementary figures and images for: MTHFD2 promotes tumorigenesis and metastasis in lung adenocarcinoma by regulating AKT/GSK‐3β/β‐catenin signalling
Source: J Cell Mol Med. 2021 Jun 13;25(14):7013–27. doi: 10.1111/jcmm.16715 (PMC8278097; doi:10.1111/jcmm.16715)

A

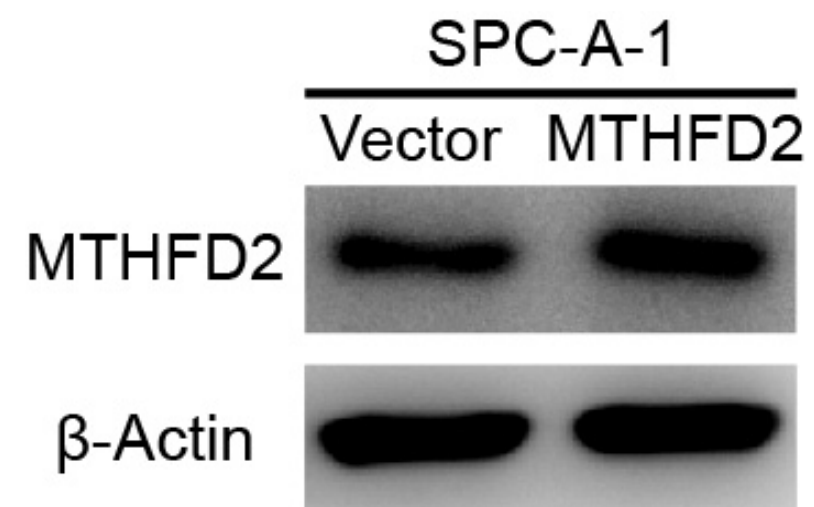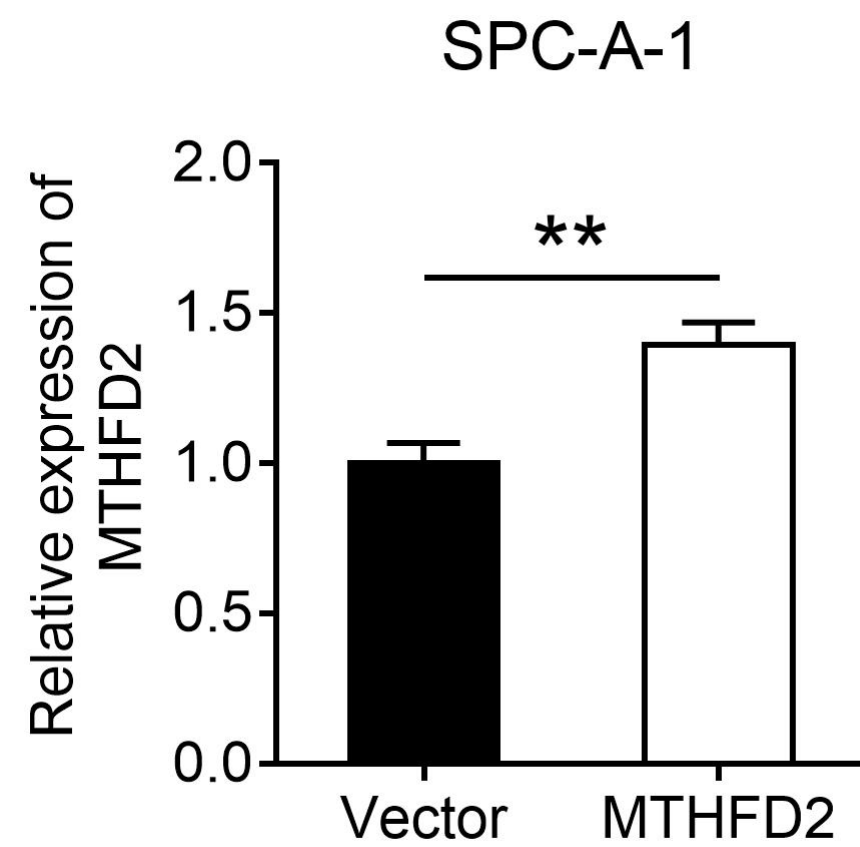

B

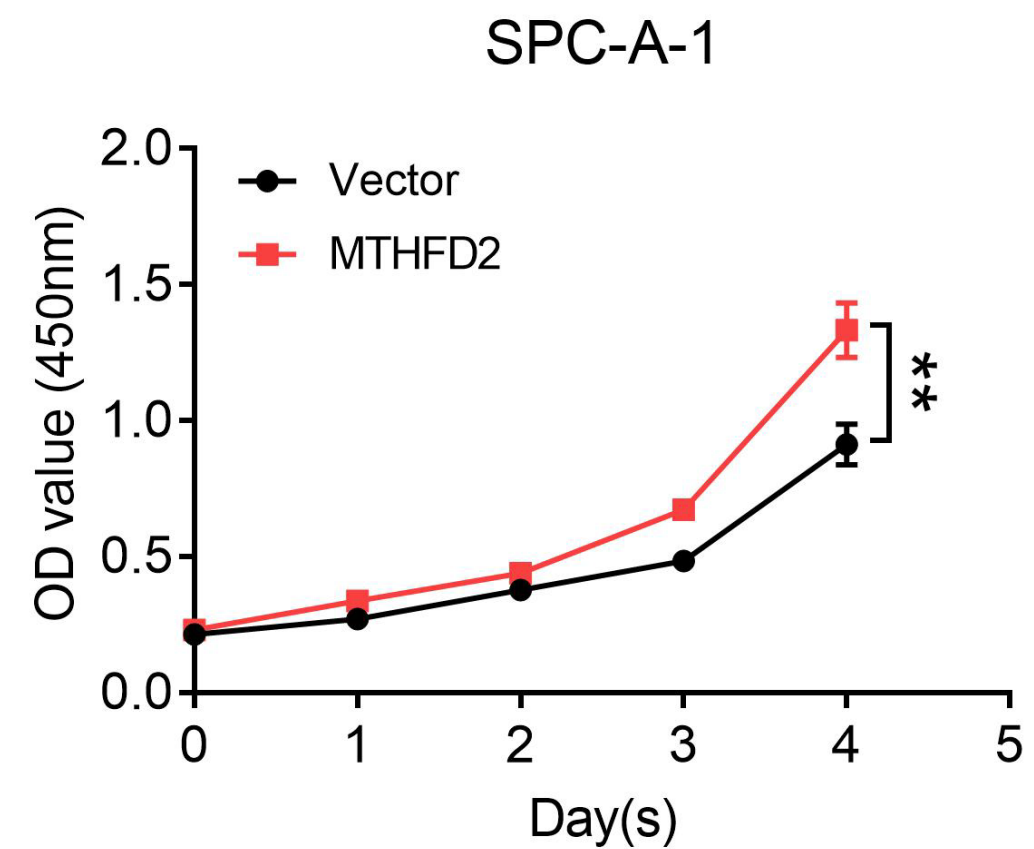

C

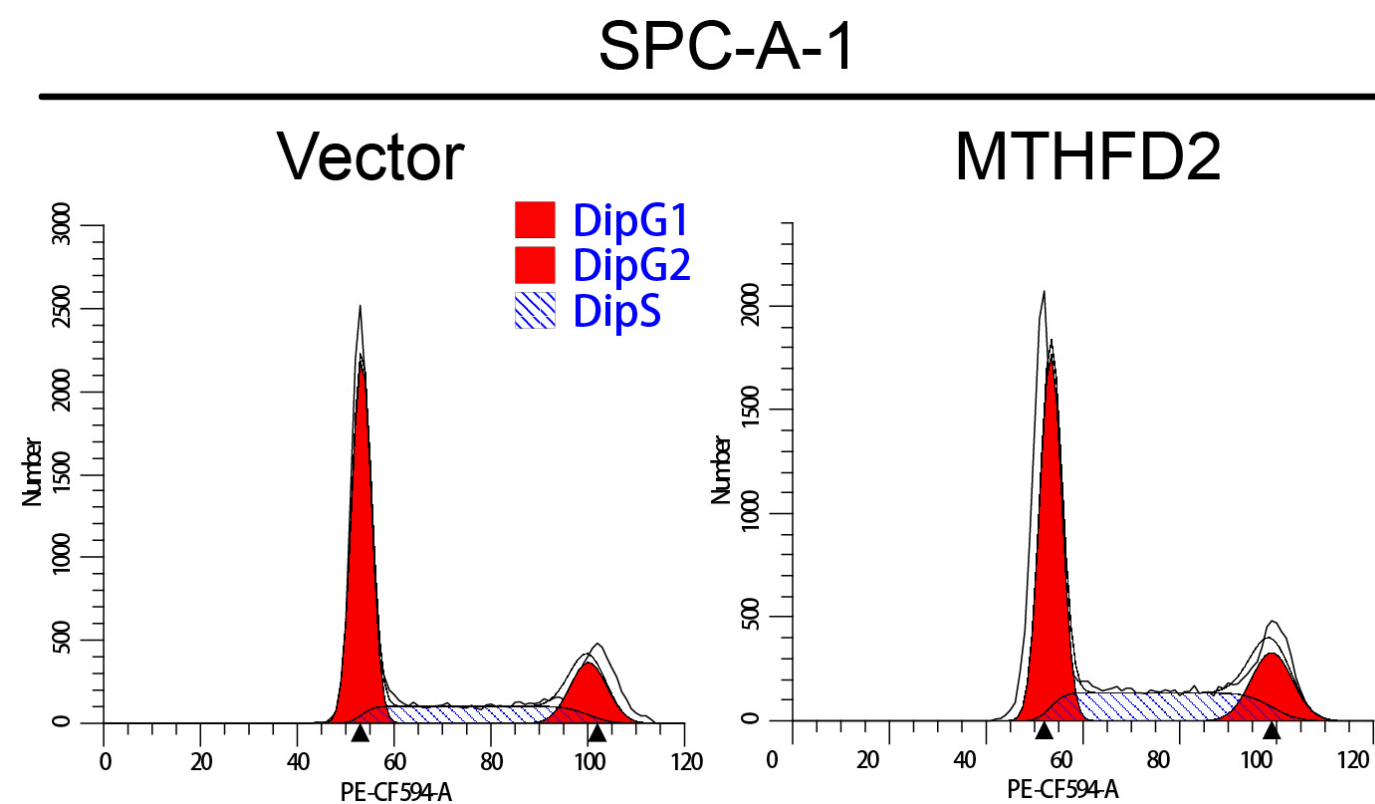

D

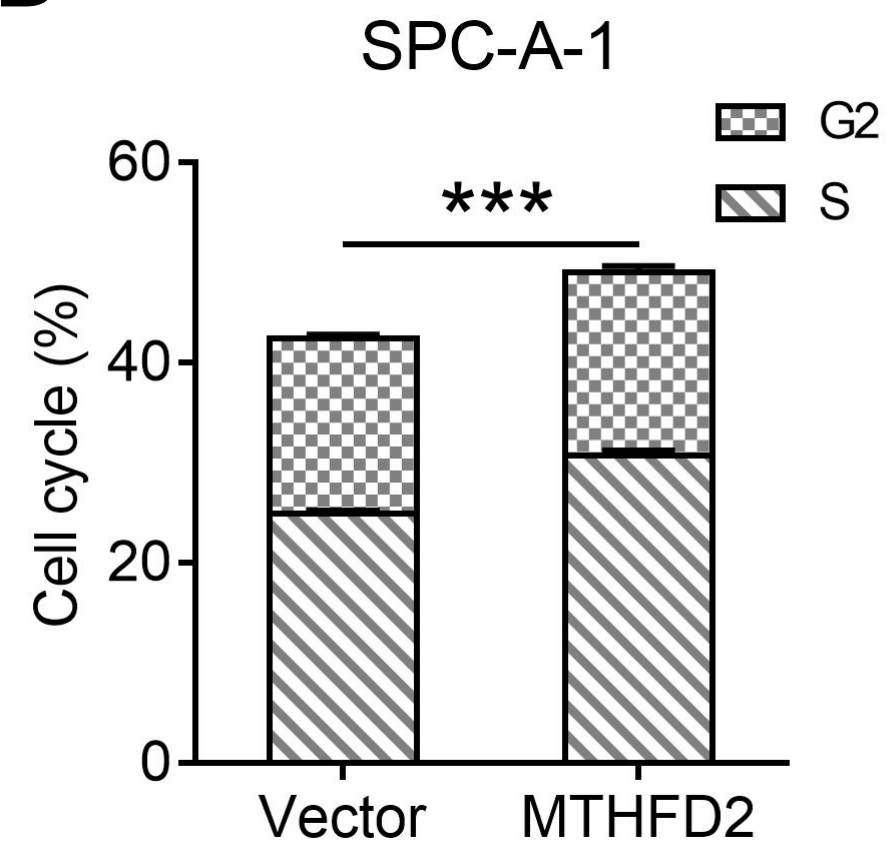

FIGURE S1

Supplement: Supplementary file 1 — Fig S1 [file JCMM-25-7013-s002.pdf]

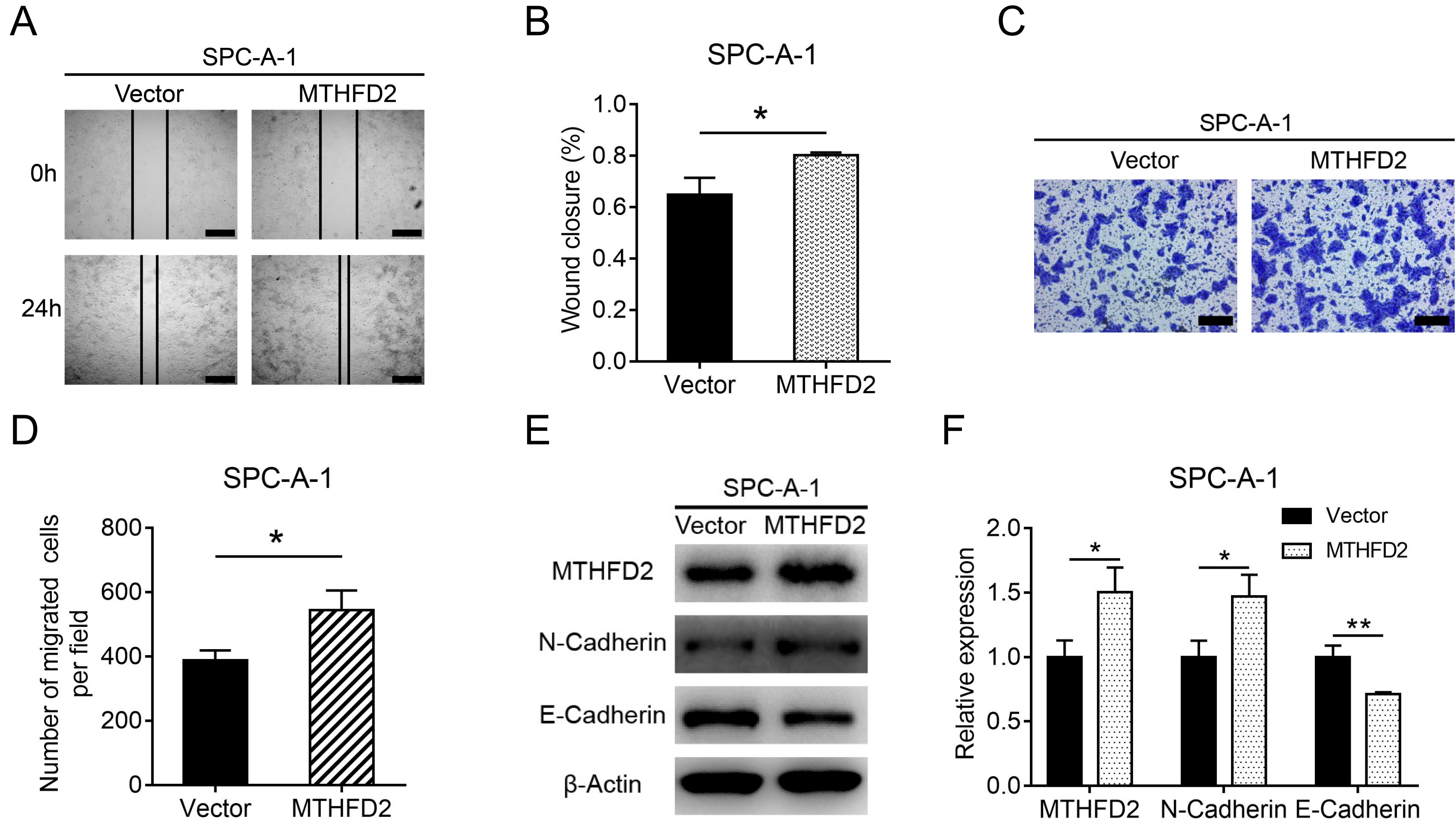

FIGURE S2

Supplement: Supplementary file 2 — Fig S2 [file JCMM-25-7013-s003.pdf]
