# Supplementary material for: MTHFD2 promotes tumorigenesis and metastasis in lung adenocarcinoma by regulating AKT/GSK‐3β/β‐catenin signalling
Source: J Cell Mol Med. 2021 Jun 13;25(14):7013–27. doi: 10.1111/jcmm.16715 (PMC8278097; doi:10.1111/jcmm.16715)
Supplement: Supplementary file 3 — Table S1 [file JCMM-25-7013-s001.docx]

| Gene | EntrezID | RefseqID | miRNA | miRWalk | miRanda | miRDB | Targetscan | SUM |
| --- | --- | --- | --- | --- | --- | --- | --- | --- |
| MTHFD2 | 10797 | NM_006636 | hsa-miR-4670-3p | 1 | 1 | 1 | 1 | 4 |
| MTHFD2 | 10797 | NM_006636 | hsa-miR-936 | 1 | 1 | 1 | 1 | 4 |
| MTHFD2 | 10797 | NM_006636 | hsa-miR-4480 | 1 | 1 | 1 | 1 | 4 |
| MTHFD2 | 10797 | NM_006636 | hsa-miR-1324 | 1 | 1 | 1 | 1 | 4 |
| MTHFD2 | 10797 | NM_006636 | hsa-miR-9-5p | 1 | 1 | 1 | 1 | 4 |
| MTHFD2 | 10797 | NM_006636 | hsa-miR-384 | 1 | 1 | 1 | 1 | 4 |
| MTHFD2 | 10797 | NM_006636 | hsa-miR-767-3p | 1 | 1 | 1 | 1 | 4 |
| MTHFD2 | 10797 | NM_006636 | hsa-miR-500a-5p | 1 | 1 | 1 | 1 | 4 |
| MTHFD2 | 10797 | NM_006636 | hsa-miR-888-5p | 1 | 1 | 1 | 1 | 4 |
| MTHFD2 | 10797 | NM_006636 | hsa-miR-1299 | 1 | 1 | 1 | 1 | 4 |
| MTHFD2 | 10797 | NM_006636 | hsa-miR-3163 | 1 | 1 | 1 | 1 | 4 |
| MTHFD2 | 10797 | NM_006636 | hsa-miR-3920 | 1 | 1 | 1 | 1 | 4 |
| MTHFD2 | 10797 | NM_006636 | hsa-miR-371a-5p | 1 | 1 | 1 | 1 | 4 |
| MTHFD2 | 10797 | NM_006636 | hsa-miR-30a-3p | 1 | 1 | 1 | 1 | 4 |
| MTHFD2 | 10797 | NM_006636 | hsa-miR-4662a-5p | 1 | 1 | 1 | 1 | 4 |
| MTHFD2 | 10797 | NM_006636 | hsa-miR-30d-3p | 1 | 1 | 1 | 1 | 4 |
| MTHFD2 | 10797 | NM_006636 | hsa-miR-4775 | 1 | 1 | 1 | 1 | 4 |
| MTHFD2 | 10797 | NM_006636 | hsa-miR-30e-3p | 1 | 1 | 1 | 1 | 4 |
| MTHFD2 | 10797 | NM_006636 | hsa-miR-577 | 1 | 1 | 1 | 1 | 4 |
| MTHFD2 | 10797 | NM_006636 | hsa-miR-548l | 1 | 1 | 1 | 1 | 4 |

Table S1. Predicted targeting MTHFD2 miRNAs from 4 databases.
